# Supplementary material for: Predictors of iron deficiency anaemia among children aged 6–59 months in Tanzania: Evidence from the 2015–16 TDHS-MIS cross-sectional household survey
Source: PLOS Glob Public Health. 2022 Nov 10;2(11):e0001258. doi: 10.1371/journal.pgph.0001258 (PMC10022048; doi:10.1371/journal.pgph.0001258)
Supplement: S1 Table — (DOCX) [file pgph.0001258.s001.docx]

| **Supporting Information**  **S1 Table**. Definition and categorisation of potential variables used in the study | |
| --- | --- |
| **Independent variables** | **Categorisation** |
| ***Household factors*** |  |
| Household wealth Index | hv217 (the household wealth index factor score) constructed by DHS based on a selected set of household assets. In quintiles, 1= richest; 2= richer; 3= middle; 4= poorer; 5= poorest) |
| Residence | In the 2 following categories:(1=urban; 2=rural) |
| Number of children under 5 years | In the 3 following categories: (1= None; 2= 1 to 2; 3= ≥3) |
| Household size | In the 2 following categories: (1= 2-5; 2= >6) |
| Source of drinking water | In the 2 following categories: (1= improved; 2=Unimproved)  piped into dwelling, piped to yard or plot, piped to neighbour, public tap or standpipe, tube well or borehole, protected well, protected spring, tanker truck and bottled water. Unimproved otherwise |
| Type of toilet facility | In the 2 following categories: (1= improved; 2=Unimproved)  Improved was flush to piped sewer system, flush to septic tank, flush to pit latrine, ventilated improved pit latrine, pit latrine with slab, and composting toilet. Unimproved otherwise. |
| ***Maternal factors*** |  |
| Maternal working status | In the 2 following categories: (1=not working; 2=working (for the past 12 months)) |
| Maternal education | In the 3 following categories: (1=Secondary or higher; 2=Primary; 3=No education) |
| Maternal age | In the 3 following categories: (1= 15-19 years; 2= 20-34 years; 3= 35-49 years) |
| Marital status | In the 2 following categories: (1= Currently married; 2=divorced/separated/widow) |
| Maternal anaemia | In the 2 following categories: (1= Non anaemic; 2= anaemic)  haemoglobin (Hb) < 110g/dl for pregnancy women |
| Maternal BMI | In the 3 following categories: (1= 25+ kg/m^2^; 2= 19-25 kg/m^2^; 3= ≤18.5kg/m^2^) |
| Mothers currently pregnant | In the 2 following categories: (1= Not at all; 2=Yes) |
| Mothers taking iron during pregnancy | In the 2 following categories: (1= Not at all; 2=Yes) |
| Combined mode and place of delivery | In the 3 following categories: (1= Vaginal; 2= Caesarean; 3= Home) |
| Type of delivery assistance | In the 2 following categories: (1=Health professional^&^; 2= non-Health professional) |
| Antenatal Clinic visits | In the 4 following categories: (1) 8+ antenatal care visits, (2) 4-7 antenatal care visits, and (3) 1-3 antenatal care visits (4) no antenatal care visits from a skilled provider for the most recent birth |
| mothers reading newspapers | In the 2 following categories: (1= At least once a week; 2= Less than once a week and 3= Never) |
| mothers listening to radio | In the 2 following categories: (1= At least once a week; 2= Less than once a week and 3= Never) |
| mothers watching television | In the 2 following categories: (1= At least once a week; 2= Less than once a week and 3= Never) |
| Power over earning | In the 2 following categories:(1=Yes; 2= No)  Yes- respondent alone, respondent and husband/partner. No- otherwise |
| Power over household decision making | In the 2 following categories:(1=Yes; 2= No)  Yes- respondent alone, respondent and husband/partner. No- otherwise |
| Autonomy over health care | In the 2 following categories:(1=Yes; 2= No)  Yes- respondent alone, respondent and husband/partner. No- otherwise |
| ***Child Factors*** |  |
| Combined birth interval and birth order | (1= 1st birth rank; 2= 2nd/3rd birth rank, more than 2 years interval; 3= 2nd/3rd birth rank, less than or equal to 2 years; 4= 4th birth rank, more than 2 years interval; 5= 4th birth rank, less than or equal to 2 years) |
| Sex of baby | In the 2 following categories: (1=Male; 2=Female) |
| Size of baby | In the 3 following categories: (1= Large; 2=Average; 3= Small) |
| Age of child (months) | In the 2 following categories: (1=6-23 months; 2=24-59 months.) |
| Child ever breastfed | In the 2 following categories: (1=Never Breastfed; 2= Ever Breastfed) |
| Minimum Acceptable Diet | In the 2 following categories: (1= No; 2=Yes/some) |
| Minimum Meal Frequency | In the 2 following categories: (1= No; 2=Yes/some) |
| Minimum Dietary Diversity | In the 2 following categories: (Yes; 1= (≥ 4 of 7 food groups) = No; 2= (< 4 of 7 food groups). |
| Dietary Diversity | In the 3 following categories: (1= High Diversity; 2= Moderate Diversity; 3= Low Diversity)  High diversity (5-7 food groups); moderate diversity (3-4 food groups) & low diversity (0–2 food groups) |
| Stunting | In the 2 following categories: (1= Not stunted; 2= stunted (height-for-age z-score < -2 SD) |
| Wasting | In the 2 following categories: (1= Not wasted; 2= wasted (weight-for-height z-score < -2 SD) |
| Underweight | In the 2 following categories: (1= Not underweight; 2= underweight (weight-for-age z-score < -2 SD) |
| Children under 5 years who slept with bed net | In the 2 following categories: (1= No; 2=Yes) |
| Children given drugs for preventing worm infestation | In the 2 following categories: (1= No; 2=Yes) |
| Children fully vaccinated | In the 2 following categories: (1= None; 2= full) |
| Vitamin A | In the 2 following categories: (1= No; 2=Yes) |
| Had diarrhoea recently | In the 2 following categories: (1= No; 2=Yes) |
| Had fever in last 2 weeks | In the 2 following categories: (1= No; 2=Yes) |

&= doctor; nurse/midwife/paramedics, family welfare visitor medical assistant/community medical officer/ health assistant
