# Supplementary material for: Predictors of iron deficiency anaemia among children aged 6–59 months in Tanzania: Evidence from the 2015–16 TDHS-MIS cross-sectional household survey
Source: PLOS Glob Public Health. 2022 Nov 10;2(11):e0001258. doi: 10.1371/journal.pgph.0001258 (PMC10022048; doi:10.1371/journal.pgph.0001258)
Supplement: Attachment — Submitted filename: Response to reviewers.doc [file pgph.0001258.s002.doc]

**A rebuttal letter that responds to each point raised by the editor and reviewer(s)**

**Response to the editor**

**Editor General Comment**: *Thank you for submitting your manuscript to PLOS Global Public Health. After careful consideration, we feel that it has merit but does not fully meet PLOS Global Public Health’s publication criteria as it currently stands. Therefore, we invite you to submit a revised version of the manuscript that addresses the points raised during the review process..*

**Response: Thank you for coordinating the review process. We appreciate the time and effort that you and the reviewers have dedicated on providing invaluable comments and suggestions on this manuscript. We have revised the manuscript and addressed reviewer’s comments.**

**Journal Requirements**

**Comment 1**: *Please* ***amend your*** ***detailed Financial Disclosure statement****. This is published with the article, therefore should be completed in full sentences, and contain the exact wording you wish to be published. If you did not receive any funding for this study, please simply state: “The authors received no specific funding for this work.”*

**Response 1: We agree with this comment and rephrase the financial disclosure statement as follows “the authors received no specific funding for this work”.**

**Comment 2**: *In the online submission form, you indicated that "All datasets are available upon request from the DHS website (https://dhsprogram.com/)". All PLOS journals now require all data underlying the findings described in their manuscript to be freely available to other researchers, either 1. In a public repository, 2. Within the manuscript itself, or 3. Uploaded as supplementary information. This policy applies to all data except where public deposition would breach compliance with the protocol approved by your research ethics board. If your data cannot be made publicly available for ethical or legal reasons (e.g., public availability would compromise patient privacy), please explain your reasons by return email and your exemption request will be escalated to the editor for approval. Your exemption request will be handled /independently and will not hold up the peer review process, but will need to be resolved should your manuscript be accepted for publication. One of the Editorial team will then be in touch if there are any issues.*

**Response 2: We agree with this comment and rephrase the statement as all datasets underlying this study are freely available at the DHS public repository ‘https://dhsprogram.com/data/dataset/Tanzania_Standard-DHS_2015.cfm?flag=0’.**

**Comment 3**: *Please provide separate figure files in .tif or .eps format only and remove any figures embedded in your manuscript file. Please ensure that all files are under our size limit of 20MB.  For more information about how to convert your figure files please see our guidelines: https://journals.plos.org/globalpublichealth/s/figures*

*Once you've converted your files to .tif or .eps, please also make sure that your figures meet our format requirements*

**Response 3: We agree with this comment and change the figures into tif format.**

**Comment 4:** *Please ensure that you refer to Figure 2 in your text as, if accepted, production will need this reference to link the reader to the figure.*

**Response 4: We agree and the text below was indicated in the manuscript last paragraph of result section- sample characteristic page 10**

**Comment 5**: *We notice that your supplementary tables are included in the manuscript file. Please remove them and upload them with the file type 'Supporting Information'. Please ensure that all Supporting Information files are included correctly and that each one has a legend listed in the manuscript after the references list.*

**Response 5: Thank you very much for pointing this out. We agree with this comment and incorporate your suggestions.**

**Reviewer's Responses to Questions**

1. **Comments to the Author** *Does this manuscript meet PLOS Global Public Health’s* [*publication criteria*](https://journals.plos.org/globalpublichealth/s/criteria-for-publication)*? Is the manuscript technically sound, and do the data support the conclusions? The manuscript must describe methodologically and ethically rigorous research with conclusions that are appropriately drawn based on the data presented.*

*Reviewer #1: Partly*

*Reviewer #2: Partly*

**Response: Thank you very much for your consideration.**

2. ***Comments to the Author***

Has the statistical analysis been performed appropriately and rigorously?

Reviewer #1: Yes

Reviewer #2: Yes

**Response: Thank you very much for your consideration.**

3. ***Comments to the Author***

*Have the authors made all data underlying the findings in their manuscript fully available (please refer to the Data Availability Statement at the start of the manuscript PDF file)?
The* [*PLOS Data policy*](https://journals.plos.org/globalpublichealth/s/data-availability) *requires authors to make all data underlying the findings described in their manuscript fully available without restriction, with rare exception. The data should be provided as part of the manuscript or its supporting information, or deposited to a public repository. For example, in addition to summary statistics, the data points behind means, medians and variance measures should be available. If there are restrictions on publicly sharing data—e.g. participant privacy or use of data from a third party—those must be specified.*

*Reviewer #1: Yes*

*Reviewer #2: No*

**Response: Thank you very much for your consideration. We agree with these comments and rephrase the statement of data availability.**

**4. *Comments to the Author***

*Is the manuscript presented in an intelligible fashion and written in Standard English?
PLOS Global Public Health does not copyedit accepted manuscripts, so the language in submitted articles must be clear, correct, and unambiguous. Any typographical or grammatical errors should be corrected at revision, so please note any specific errors here.*

*Reviewer #1: Yes*

*Reviewer #2: Yes*

**Response: Thank you very much for your consideration**

**Review Comments to the Author**

**Reviewer #1:** No doubt the paper is of public health importance. But, there must be some mention and findings regarding:

1. Feeding pattern of infants, especially whether breast-fed or not.

**Response: Thank you very much for pointing this out. Exploring whether infants are breast-fed or not is another research question of interest and should be considered as another important manuscript in the research area. However, feeding pattern of infants is relevant to children aged 6-23 months and we found in this study that MAD, MMF and MMD did not differ statistically significant in the bivariate analysis. However, after 6 months, the mother’s milk alone becomes nutritionally insufficient for the child, therefore nutritionally adequate and safe Complementary Feeding from the 6th month is recommended for better health and development of the infant.**

2. Exclusion of family history of Thalassemia and sickle-cell anemia*.*

**Response: Thank you very much for pointing this out. We agree with this argument. This is the one of our study limitations as well as future research in last paragraph of the discussion section.**

3. Inclusion and exclusion criteria of study population

**Response: Thank you very much for pointing this out. The inclusion criteria of this study were children aged 6–59 months and lived with the respondent (women aged 15-49 years) as indicated in page 6, paragraph 1.**

**Reviewer #2**: this study intended to assess factors associated with anaemia among children aged 6-59 months and analysed maternal, child and household various factors which all related to iron deficiency anaemia. But to find out the actual picture of anaemia, it is very important to rule out thalassemia, sickle cell disease, haemoglobinopathies and carrier status of mother and child. So, the study data is not sufficient to support the conclusions.
Supplied link does not specify the data underlying the findings described in the manuscript.

**Response: Thank you very much for pointing this out. We agree with this argument. This is the one of our study limitations as well as future research in last paragraph of the discussion section.**
