# Supplementary material for: Predictors of iron deficiency anaemia among children aged 6–59 months in Tanzania: Evidence from the 2015–16 TDHS-MIS cross-sectional household survey
Source: PLOS Glob Public Health. 2022 Nov 10;2(11):e0001258. doi: 10.1371/journal.pgph.0001258 (PMC10022048; doi:10.1371/journal.pgph.0001258)
Supplement: Attachment — Submitted filename: A rebuttal letter2_final.docx [file pgph.0001258.s003.docx]

Dear Faisal Abbas, PhD,

Once again, thank you very much for giving us the opportunity to submit a second revised draft of our manuscript titled “Predictors of anaemia among children aged 6–59 months in Tanzania: evidence from the 2015-16 TDHS-MIS cross-sectional household survey” to *PLOS Global Public Health*.

We appreciate the time and effort that you and reviewers have dedicated to providing valuable feedback on our manuscript.

Here is a point-by-point response to the editor and reviewers’ comments and concerns.

**Comment 1:** *If the authors have adequately addressed your comments raised in a previous round of review and you feel that this manuscript is now acceptable for publication, you may indicate that here to bypass the “Comments to the Author” section, enter your conflict of interest statement in the “Confidential to Editor” section, and submit your "Accept" recommendation.*

*Reviewer #1: All comments have been addressed*

*Reviewer #2: (No Response).*

**Response**: We highly appreciate comment from *Reviewer#1* and, we have carefully edited the manuscript according to the inputs from the two reviewers. We truly hope that the revised manuscript is clear enough to follow.

**Comment 2:** *Does this manuscript meet PLOS Global Public Health’s* [*publication criteria*](https://journals.plos.org/globalpublichealth/s/criteria-for-publication)*? Is the manuscript technically sound, and do the data support the conclusions? The manuscript must describe methodologically and ethically rigorous research with conclusions that are appropriately drawn based on the data presented.*

*Reviewer #1: Yes*

*Reviewer #2: Partly*

**Response:** Thank you very much for this comment and, in responding to *Reviewer#2,* the methods section have been carefully rewritten and reorganized to enhance the logic flow (line number 122-186).

**Comment 3:** *Has the statistical analysis been performed appropriately and rigorously?*

*Reviewer #1: I don't know*

*Reviewer #2: Yes*

**Response:** Thank you very much for this comment and, in responding to *Reviewer#1,*we carefully add table 5 that described variables used in this study and enhance the main messages they are supposed to convey to the readers.

**Comment 4:** *Have the authors made all data underlying the findings in their manuscript fully available (please refer to the Data Availability Statement at the start of the manuscript PDF file)?
The* [*PLOS Data policy*](https://journals.plos.org/globalpublichealth/s/data-availability) *requires authors to make all data underlying the findings described in their manuscript fully available without restriction, with rare exception. The data should be provided as part of the manuscript or its supporting information, or deposited to a public repository. For example, in addition to summary statistics, the data points behind means, medians and variance measures should be available. If there are restrictions on publicly sharing data—e.g. participant privacy or use of data from a third party—those must be specified.*

*Reviewer #1: Yes*

*Reviewer #2: Yes*

**Response:** Thank you very much and, we highly appreciate the reviewers’ insightful and helpful comments on our manuscript.

**Comment 5:** *Is the manuscript presented in an intelligible fashion and written in standard English?
PLOS Global Public Health does not copyedit accepted manuscripts, so the language in submitted articles must be clear, correct, and unambiguous. Any typographical or grammatical errors should be corrected at revision, so please note any specific errors here.*

*Reviewer #1: Yes*

*Reviewer #2: Yes*

**Response**: Thank you very much and, we highly appreciate the reviewers’ insightful and helpful comments on our manuscript.

**Comment 6**: *Review Comments to the Author
Please use the space provided to explain your answers to the questions above. You may also include additional comments for the author, including concerns about dual publication, research ethics, or publication ethics. (Please upload your review as an attachment if it exceeds 20,000 characters)*

*Reviewer #1: May be published.*

*Reviewer #2: Authors totally ignored the comments raised in previous round of review. Hemolytic anemia such as thalassemia and other hemoglobinopathies must be find out or excluded to evaluate the early childhood anaemia and the risk factors. So, I feel that, this manuscript is not acceptable for publication.*

**Response:** Thank you very much for pointing this out. In responding to *Reviewer #2,* first this paper is the secondary analysis of data collected in the Tanzania Demographic and Health Survey (TDHS) 2015-16. However, Hemolytic anemia such as thalassemia and other hemoglobinopathies were not assessed and, this was stated a limitation of this paper (lines 383-388). Second, we agreed with *Reviewer #2* comment ‘*Hemolytic anemia such as thalassemia and other hemoglobinopathies must be find out or excluded to evaluate the early childhood anaemia and the risk factors’* and, we have carefully edited the manuscript according to her/his inputs (lines 95- 105; lines 110- 114; lines 383- 388 and; lines 399- 400). We also add two references 50 and 51. Third, we have carefully edited the manuscript according to the inputs from the two reviewers. We truly hope that the revised manuscript is clear enough to follow. We highly appreciate Reviewer#2’s insightful comments on improving the paper.

Sincerely,

Ray Masumo, PhD
