# Supplementary material for: Predictors of iron deficiency anaemia among children aged 6–59 months in Tanzania: Evidence from the 2015–16 TDHS-MIS cross-sectional household survey
Source: PLOS Glob Public Health. 2022 Nov 10;2(11):e0001258. doi: 10.1371/journal.pgph.0001258 (PMC10022048; doi:10.1371/journal.pgph.0001258)
Supplement: Attachment — Submitted filename: rose- rebuttal letter_2022_08_09.docx [file pgph.0001258.s004.docx]

Dear Dr. Marianella Herrera-Cuenca,

Thank you very much for giving us the opportunity to submit a third revised draft of our manuscript titled “Predictors of anaemia among children aged 6–59 months in Tanzania: evidence from the 2015-16 TDHS-MIS cross-sectional household survey” to *PLOS Global Public Health*.

We appreciate the time and effort that you and reviewers have dedicated to providing valuable feedback on our manuscript.

Here is a point-by-point response to the editor and reviewers’ comments and concerns.

**Review Comments to the Author**

**Comment 1***: Is umbilical cord ligation after 30 seconds to 3 minutes a universal practice? Recognizing its impact on iron stores early in childhood, it can bias the result in the population studied*.

**Response**: Thank you very much and, we highly appreciate the reviewers’ insightful and helpful comments on our manuscript. In line numbers 383-385, in the study limitation, we have rewritten that section and indicated that ‘*despite the WHO recommendation in 2014 on delayed umbilical cord clamping and cutting to reduce the risk of anaemia and hypoxia in infants, however, its implementation is still low in many countries including Tanzania*’

To support the above mentioned statement, we have added two references (line number 573- 578) i.e.

*WHO. Guideline: Delayed umbilical cord clamping for improved maternal and infant health and nutrition outcomes. Geneva World Health Organization. 2014 [cited 2022/08; Available from: http://apps.who.int/iris/bitstream/handle/10665/148793/9789241508209_eng.pdf.*

*Mwakawanga DL, Mselle LT. Early or delayed umbilical cord clamping? Experiences and perceptions of nurse-midwives and obstetricians at a regional referral hospital in Tanzania. PLoS One. 2020; 15:e0234854.*

Sincerely,

Ray Masumo, PhD
