# Supplementary material for: Predictors of iron deficiency anaemia among children aged 6–59 months in Tanzania: Evidence from the 2015–16 TDHS-MIS cross-sectional household survey
Source: PLOS Glob Public Health. 2022 Nov 10;2(11):e0001258. doi: 10.1371/journal.pgph.0001258 (PMC10022048; doi:10.1371/journal.pgph.0001258)
Supplement: Attachment — Submitted filename: A rebuttal letter2_Rose_04_10_2022.docx [file pgph.0001258.s005.docx]

Dear Marianella Herrera-Cuenca, MD, PhD

Once again, thank you very much for giving us the opportunity to submit a revised draft of our manuscript titled “Predictors of anaemia among children aged 6–59 months in Tanzania: evidence from the 2015-16 TDHS-MIS cross-sectional household survey” to *PLOS Global Public Health*.

We appreciate the time and effort that you have dedicated to providing valuable feedback on our manuscript.

Here is a response to the editor’ concerns.

Comments: *This is a very important topic for public health however you need to consider some major revisions such as the title of the article: did you address all micronutrients related to anemia? No, then why don’t you make some more specific such as ferropenic anemia (just an idea) please check carefully all revisors comments and work on them*

**Response**: We highly appreciate the insightful and helpful comments on our manuscript.

Thank you very much!

We have carefully rewritten the title (line 2), abstract (lines 36-60), introduction (lines 67-116), methods (lines 137- 200), results (lines 249- 278), discussion (lines 282- 359) and conclusion (lines 401-403).

We genuinely hope that the revised manuscript is much clear and more explicit in reporting iron deficiency anaemia in Tanzania

Sincerely,

Ray Masumo, PhD
